# Molecular Characterization of DENV‐1 Isolates From Pakistan Reveals Immunogenic Variability and Antiviral Potential of Phytochemicals Targeting the CprM Region

**DOI:** 10.1155/jotm/9932618

**Published:** 2026-08-30

**Authors:** Sajid Ali Shah, Syed Ahsan Shahid, Sidra Rahman, Muhammad Ali

**Affiliations:** ^1^ Department of Biotechnology, Quaid-I-Azam University, Islamabad, Pakistan, qau.edu.pk; ^2^ Natural and Medical Sciences Research Center, University of Nizwa, Birkat Al Mouz, Nizwa, Oman, unizwa.edu.om; ^3^ Institute of Pathology and Diagnostic Medicine, Khyber Medical University, Peshawar, Pakistan, kmu.edu.pk

**Keywords:** ADMET profiling, CprM gene, dengue virus (DENV), DENV outbreak, DENV-1, molecular docking, phylogenetic analysis

## Abstract

Dengue virus (DENV), a mosquito‐borne arbovirus, remains a critical global health concern, causing approximately 390 million infections annually. Dengue fever poses significant challenges to healthcare systems in endemic regions like Pakistan due to the presence of four established serotypes (DENV‐1–DENV‐4) and the potential emergence of a fifth serotype (DENV‐5). The current study aimed to investigate molecular characteristics of DENV‐1 isolates from Peshawar, Pakistan, focusing on serotyping, genetic variability in the CprM junction region, and identification of potential antiviral phytochemicals. A total of 45 patient samples were screened, with the highest infection rate (49%) observed among individuals aged 20–40 years. Phylogenetic analysis revealed that six isolates clustered within the DENV‐1 clade and grouped closely related to isolates from China, India, Bangladesh, and Pakistan, suggesting a regional evolution and potentially cross‐border transmission. The prominent amino acid substitutions were Q47H, G48E, P49H, K51 E/N, L52 M, and M57I, which may influence viral structure, immune evasion, and antigenic properties. To address the lack of effective antivirals, a library of 60 phytochemicals was screened. Nine compounds demonstrated favorable ADMET profiles and high binding affinity (docking scores < −100) with the CprM region. Silydianin exhibited the strongest interaction (−159.81), indicating promising inhibitory potential. This integrative molecular and computational approach enhances our understanding of circulating DENV‐1 strains and highlights plant‐derived compounds as candidates for future antiviral development, pending in vitro validation.

## 1. Introduction

Dengue virus (DENV) is a mosquito‐borne, positive‐sense RNA virus belonging to the family Flaviviridae, causing varying degrees of illness from mild to life‐threatening dengue hemorrhagic fever (DHF) and dengue shock syndrome (DSS) [1]. The main vectors of DENV transmission to human hosts are *Aedes aegypti* and, to some extent, *Aedes albopictus* mosquitoes [2]. There are four antigenic variants of DENV: DENV‐1–DENV‐4, which have worldwide recognition; though there has been evidence of another antigenic form, DENV‐5; its role in human epidemiology has not been clearly elucidated [3]. The error‐prone RNA‐dependent RNA polymerase of DENV contributes to high adaptability and rapid evolution of this virus, leading to immune evasion and different severities of disease [4]. There have been no effective antiviral drugs discovered so far, and existing anti‐DENV vaccines vary in efficacy depending on antigenic variants [5, 6].

Pakistan has been reported to remain hyperendemic with respect to dengue, with almost annual outbreaks and a dynamic pattern of dominant serotypes [7]. Analysis of the recent 2024 outbreak in the region, using whole‐genome sequencing, indicated the cosmopolitan genotype of DENV‐2 as the dominant serotype (accounting for 93% of PCR‐positive cases), along with the coexistence of DENV‐1, genotype III, with a high degree of genomic similarity to the strains of previous years, thus indicating the possibility of ongoing local transmission, the existence of a lineage, and highlighting shifts in serotype prevalence [8]. Mutational analysis has indicated the existence of regional adaptations, possibly influencing the viral fitness and transmission dynamics. The longitudinal serotype study in the region, conducted over the previous decade, has further supported the cyclic changes in the dominant serotype in the regional clinical centers, thus emphasizing the dynamic nature of the dengue epidemiology in the region of Pakistan [9]. Reports of the independent studies in the cities of Lahore and Peshawar have indicated the important role of DENV‐1 and the coexistence of multiple serotypes in the outbreaks, thus emphasizing the importance of fine‐scale molecular mapping to inform public health responses [10].

Despite the fact that DENV‐2 has often been documented as the major contributor to major outbreaks and, for some genotypes, has shown increased vector transmission capabilities and epidemic properties, the phenomenon of serotype‐specific transmission is highly dependent on the viral genotype, community immunity, and the competence of the vectors [11, 12]. Notably, however, DENV‐1 still has major epidemiological and medical relevance, given the fact that it cocirculates in Pakistan together with DENV‐2, and there are documented differences in the clinical manifestations and severity of the disease according to the serotype, with several studies showing a considerable disease burden due to infections with DENV‐1 [13–15]. Thus, the molecular characterization of Dengue fever virus DENV‐1 remains highly relevant. Pakistan still lacks detailed sequence analysis for the regions of choice for serotyping and phylogenetic analysis, such as the CprM gene. In addition, mutations within structural junction regions could potentially affect the antigenicity and immunogenic properties; therefore, these aspects constitute the major focus of the current study.

Besides epidemiological growth, dengue disease control is further complicated by the interplay between genetic variability of the virus, host immune systems, and transmission intensity mediated by the vectors [16]. Genetic variability within the structural parts of the virus may affect virion particle formation, membrane fusion rates, and immune system recognition. The region of fusion between the capsid and premembrane protein of DENV is of special importance because this site is widely utilized for virus serotyping and genetic analysis. Genetic variation at this site may indicate prevailing changes of virus lineages within endemic areas [17]. Nevertheless, the lack of specific DENV therapies has spurred research into plant compounds as potential antidengue drugs. In silico screening with drug‐likeness, ADMET analysis, and molecular docking is a cost‐effective strategy for prioritizing plant compounds for experimental analysis [18].

However, the situation in Pakistan is further worsened by the presence of *Aedes* mosquitoes in urban areas due to the country’s climate, thereby leading to increased transmission risk associated with dengue infection. Although dengue is known to be endemic in Pakistan, with a large number of cases being reported every year [19], The scope of molecular surveillance is limited in the country, especially in the case of the CprM gene junctions. The targeted sequencing of the CprM gene can be very useful in improving the resolution of the DENV serotypes, which is very important in tracking the DENV during an outbreak and the development of vaccine strategies. The objectives of the study include (i) the amplification and sequencing of the CprM junction in the DENV strains isolated from the outbreak in the city of Peshawar, (ii) to confirm the circulating serotype in our outbreak samples by analyzing the phylogenetic relationship and amino acid variations in the protein sequences. Additionally, (iii) in silico screening of phytochemical libraries was done to identify potential antiviral drugs targeting the CprM protein of the DENV by using the molecular docking method.

## 2. Materials and Methods

**FIGURE 1:**
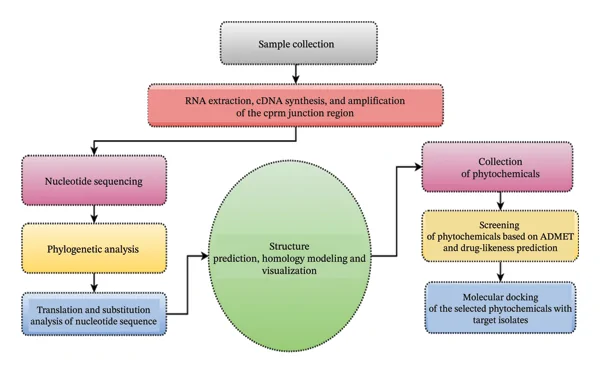
Overview of the study workflow, from sample processing and sequence analysis to structure modeling and phytochemical docking.

Current research was performed in the Infectious Diseases and Molecular Pathology Laboratory (IDMPL), Department of Biotechnology, Quaid‐i‐Azam University, Islamabad. A series of experiments were conducted under controlled conditions. A schematic representation of the methodology is shown in Figure 1.

### 2.1. Sample Collection

Blood samples from dengue‐positive patients were collected in the Peshawar district of Khyber Pakhtunkhwa Province (KP) from July to August 2021, which included patients with mild, moderate, and severe disease, as all categories were present during the outbreak. A total of 45 dengue‐positive samples were collected, of which 25 were males and 20 were females. The details are given in Table 1. All participants provided written informed consent before sample collection. The samples tested positive for dengue‐specific IgG, IgM, and NS1 antibodies. The samples were tested for dengue‐specific NS1 antigen and IgM/IgG antibodies using commercially available rapid diagnostic test (RDT) kits according to the manufacturer’s instructions. For each test, the required volume of serum was added to the sample well, followed by the assay buffer. Results were recorded after the recommended incubation time based on the appearance of colored bands in the control and test regions. Samples collected during the early phase of infection were mainly used for NS1 antigen detection, while samples collected at later stages were used for IgM and IgG antibody detection.

**TABLE 1:** Demographic information of the patients enrolled in the study.

Age group	Male	Female	Total
1–20 years	10	6	16
20–40 years	12	10	22
40–60 years	3	4	7
Total	**25**	20	**45**

*Note:* The bold values represent the subtotals for each age group and the overall totals.

### 2.2. RNA Extraction, cDNA Synthesis, and Amplification of the CprM Junction Region

Viral RNA was extracted from the serum of dengue‐positive blood samples using WizPrep Total RNA Mini Kit, following the manufacturer’s standard protocol. Complementary DNA (cDNA) was synthesized from the extracted RNA using the RevertAid cDNA Synthesis Kit (Thermo Scientific). Polymerase chain reaction (PCR) was performed for the amplification of the conserved CprM junction region using universal primers. The PCR was conducted in a 20 μL reaction volume containing 10 μL of 2X Phusion HF master mix (Thermo Scientific), 1.5 μL of D1F (TCAATATGCTGAAACGCGWGAGAAACCG), 1.5 μL of D1R (TTGCACCARCARTCWATGTCTTCWGGYTC) [20], 1 μL of ddH_2_O, and 6 μL template. The amplified PCR products were analyzed using 1.5% agarose gel electrophoresis to confirm product size and quality.

### 2.3. Nucleotide Sequence Analysis

Out of the 45 samples positive for dengue, 20 PCR products were subjected to Sanger sequencing, and the resulting nucleotide sequences were visualized through BioEdit software, and only six isolates with high‐quality and interpretable results were retained. Sequences were exported in FASTA format for downstream analyses [21]. To assess sequence similarity and identify closely related strains, a basic local alignment search tool (BLAST) analysis was performed (http://web.ncbi.nlm.gov/blast), and reference sequences were retrieved.

### 2.4. Phylogenetic Analysis

For the construction of a phylogenetic tree, reference sequences (*n* = 42) representing all four DENV serotypes (DENV‐1–DENV‐4) from different regions of the world, including Pakistan, were retrieved from NCBI GeneBank. The selected sequences were aligned through the MEGA 12 software package [22]. The phylogenetic analysis was conducted using the neighbor‐joining method with 1000 bootstrap replicates to assess the evolutionary relationships between the local isolates and the reference sequences.

### 2.5. Substitution Analysis of Amino Acid Sequences

The CprM nucleotide sequences, obtained in FASTA format, were translated into their corresponding amino acid sequences using the ExPASy Translate Tool (http://web.expasy.org/translate/) [23]. Each sample’s translated protein sequence was then aligned with the reference DENV‐1 polyprotein sequence using CLC Main Workbench software. Amino acid substitutions relative to the reference sequence were identified and documented for further analysis.

### 2.6. Structure Prediction, Homology Modeling, and Visualization

To predict the secondary structure of the CprM region of all the isolates and reference sequences, an online server and a self‐optimized prediction method (SOPMA), as well as PSIPRED (http://bioinf.cs.ucl.ac.uk/psipred/) were used. SOPMA has been described to improve the success rate in the prediction of the secondary structure of proteins [24]. The Robetta server (http://robetta.bakerlab.org) was used for the tertiary structure modeling with high accuracy. The template structure of a particular amino acid sequence is identified by the Robetta server via comparative modeling, which is accomplished using PSI‐BLAST, BLAST, FFAS03, or 3D‐Jury [25]. The three‐dimensional structure of *t* our isolates was validated using the online tools PROCHECK (https://saves.mbi.ucla.edu/). PROCHECK was used to generate the Ramachandran plot for stereochemical assessment, while ERRAT was used to evaluate the overall structural quality based on nonbonded interactions. These analyses confirmed the reliability of the predicted model for further studies [26–28]. The best 3D model was carefully chosen and saved as a PDB file for future investigation. The molecular visualization tool PyMOL (https://pymol.org/2/) was utilized to visualize and compare structures.

### 2.7. Retrieval of Phytochemicals

A total of 60 phytochemicals were selected through an extensive literature survey. Compounds were identified based on their reported antibacterial, antimicrobial, antifungal, antitumor, and antioxidative properties. The chemical structures of these phytochemicals were retrieved from the PubChem database repository (https://pubchem.ncbi.nlm.nih.gov/) and evaluated for their potential antiviral activity against the DENV [29].

### 2.8. Prediction and Screening of Phytochemicals Based on ADMET and Drug‐Likeness

The phytochemicals were filtered based on ADMET properties and drug‐likeness prediction, using the SwissADME server (http://www.swissadme.ch/index.php). The phytochemicals’ pharmacological and pharmacokinetic characteristics, including their solubility (ESOL), gastrointestinal (GI) absorption, blood–brain barrier (BBB) penetration, and violations of Lipinski’s principles, were evaluated. The cytotoxicity and carcinogenicity were also evaluated through the ProTox 3.0 online server (https://tox.charite.de/protox3/index.php?site=faq). The criteria set for screening compounds was Lipinski’s violations = 0; solubility = high; GI absorption = high or moderate; BBB permeability = no; and toxicity = zero/nil.

### 2.9. Molecular Docking of the Selected Phytochemicals With Target Isolates

The 3D SDF structures of phytochemicals selected based on their ADMET properties were retrieved from the PubChem database and converted to PDB format using PyMOL. Molecular docking was then performed using the HDOCK server (http://hdock.phys.hust.edu.cn/), and the resulting protein–ligand interactions were visualized using Discovery Studio.

## 3. Results

**FIGURE 2:**
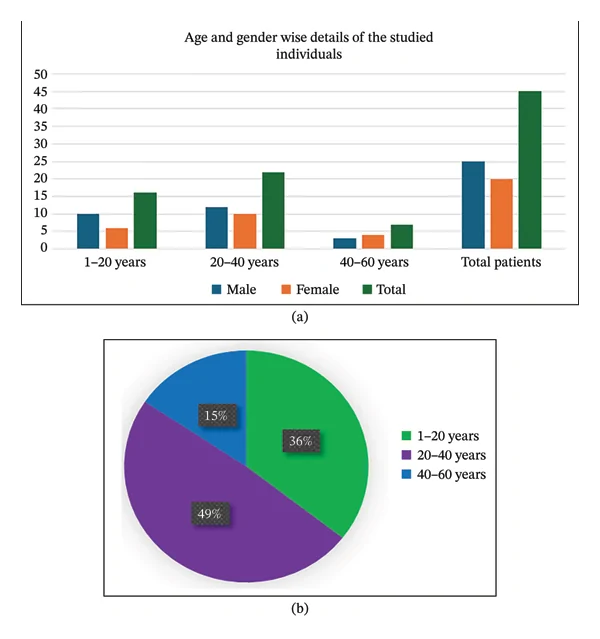
(a) Age‐ and gender‐wise distribution of dengue‐infected individuals. The bar graph illustrates the demographic breakdown of the studied population across three age groups (1–20, 20–40, and 40–60 years), stratified by gender. The total number of patients in each group is also indicated. Most cases were observed in the 20–40 years age group, with a relatively higher number of male patients reported across all age categories. (b) Age‐wise percentage distribution of dengue‐infected individuals. The pie chart presents the proportion of infected individuals within each age group. The highest infection rate (49%) was recorded among individuals aged 20–40 years, followed by 1–20 years (36%), and 40–60 years (15%), indicating an increased prevalence of infection among younger adults.

In this study, a total of 45 dengue‐positive samples were collected from District Peshawar, Pakistan. The participants included 25 males and 20 females, representing various age groups. The age and gender distribution of the patients are illustrated in Figure 2a, while Figure 2b presents the age‐wise percentage distribution. RDT analysis of patient serum samples demonstrated the presence of dengue NS1 antigen, IgM antibodies, and IgG antibodies in a proportion of the tested samples. NS1 positivity was more frequently observed in samples collected during the early phase of infection, while IgM antibodies were predominantly detected during the acute/recent phase. IgG antibodies were identified mainly in later‐stage or secondary infection cases.

### 3.1. Results of PCR‐Amplified Products

**FIGURE 3:**
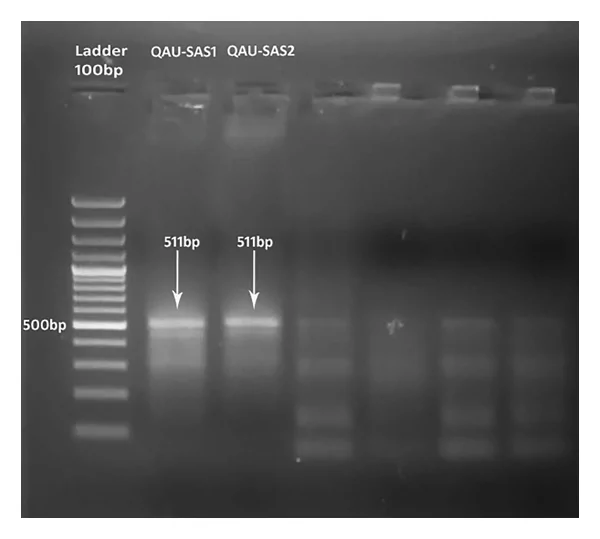
Gel electrophoresis and visualization of PCR‐amplified products. Lane 1: 100 bp DNA ladder; Lane 2 and Lane 3: amplified products from QAU‐SAS1 and QAU‐SAS2, respectively.

Following PCR amplification, the products were analyzed on a 1.5% agarose gel to confirm successful amplification. The samples were labeled as QAU‐SAS1 and QAU‐SAS2. The amplified products showed bands of approximately 511 base pairs (bp) in all samples, consistent with the expected size when compared against the 100 bp DNA ladder, as illustrated in Figure 3.

### 3.2. Phylogenetic Analysis

**FIGURE 4:**
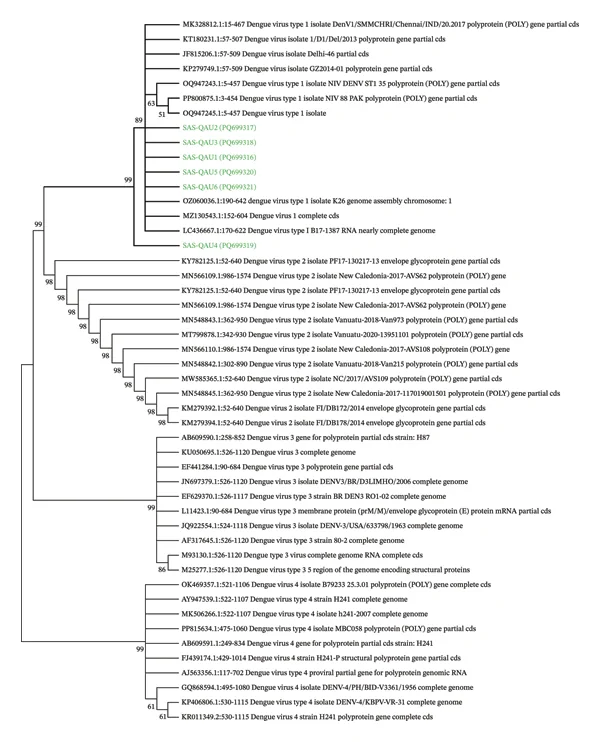
Phylogenetic tree of the CprM gene sequences from six DENV‐1 isolates (SAS‐QAU1–SAS‐QAU6, highlighted in green) in comparison with global dengue virus reference sequences. The phylogenetic analysis was conducted using the neighbor‐joining method with 1000 bootstrap replicates in MEGA 12 to assess the evolutionary relationships between the local isolates and 42 reference sequences (shown in black) retrieved from GenBank. Bootstrap values are indicated at branch nodes to reflect clustering confidence. The green‐highlighted isolates cluster closely with other DENV‐1 strains, suggesting regional genetic similarity and possible shared ancestry. This analysis provides insight into the genetic relatedness and divergence of circulating dengue virus strains.

To investigate genetic variability, the CprM gene junction of six DENV‐1 isolates (SAS‐QAU1 to SAS‐QAU6) was sequenced. A total of 42 reference sequences representing all four DENV serotypes were retrieved from the NCBI GenBank database. Phylogenetic trees were constructed using the neighbor‐joining method in MEGA 12 [30], with the Jukes–Cantor substitution model [31] that were selected based on model selection tool, which evaluates alternative nucleotide substitution models based on likelihood scores; this analysis indicated suitability for our dataset due to the relatively short sequence length and low divergence among isolates. In total, 48 nucleotide sequences were included in the analysis. Phylogenetic analysis demonstrated that the study isolates clustered together in a monophyletic group with strong bootstrap support (99%), indicating close genetic relatedness and probable regional circulation within the DENV‐1 lineage and shared common ancestor. Current isolates (SAS‐QAU1‐SAS‐QAU6) grouped closely with isolates having accession numbers OQ947245, PP800875, OQ947243, OZ060036, MZ130543, KP279749, JF815206, KT180231, MK328812, and LC436667, which were previously reported from Guangzhou (China), India, Bangladesh, and Pakistan, confirming their placement within the established DENV‐1 lineage rather than a distinct emerging clade, as illustrated in Figure 4.

### 3.3. Amino Acid Substitutions in the CprM Region of DENV1

**FIGURE 5:**
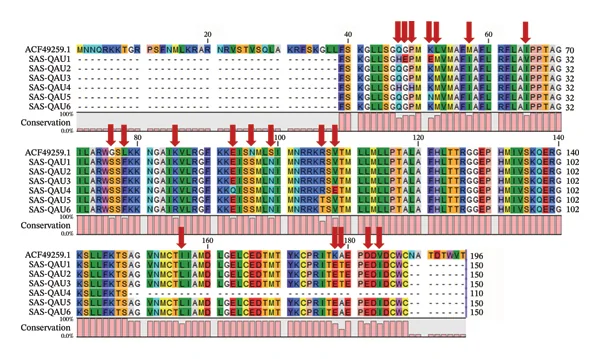
Multiple sequence alignment of amino acid sequences from DENV‐1 isolates (SAS‐QAU1–SAS‐QAU6) with the reference protein (ACF49259.1). The alignment highlights conserved and substituted residues across the sequences. Red arrows indicate the positions of amino acid substitutions observed in isolates compared to the reference protein. Colored letters represent amino acid residues based on physicochemical properties (e.g., hydrophobic, polar, charged), and the conservation bar at the bottom depicts the degree of residue conservation at each alignment position. Higher bar heights and darker shades indicate higher conservation. This alignment helps visualize sequence variation potentially impacting protein structure and function among the studied isolates.

The CprM nucleotide sequences were translated into amino acid sequences by using the ExPASy translate tool, followed by alignment of the resulting amino acid sequences with the reference protein sequence through the CLC main workbench (Version 8), as shown in Figure 5. The numbering of the amino acids is according to the reference sequence ACF49259.1 polyprotein dengue viral isolate.

The reference polyprotein sequence ACF49259.1 for DENV‐1 was retrieved from the NCBI GenBank. This sequence was aligned with the query sequences of the six studied isolates (SAS‐QAU1–SAS‐QAU6) to identify amino acid substitutions. The positions and corresponding substitutions observed in each isolate relative to the reference sequence are detailed in Table 2.

**TABLE 2:** Amino acid substitutions in the isolates (SAS‐QAU1, SAS‐QAU2, SAS‐QAU3, SAS‐QAU4, SAS‐QAU5, SAS‐QAU6) proteins as compared to the reference sequence ACF49259.1 polyprotein [dengue virus type 1].

Amino acid position^∗^	Reference sequence (ACF49259.1)	QAU‐SAS 1	QAU‐SAS 2	QAU SAS 3	QAU SAS 4	QAU SAS 5	QAU SAS 6
47	Q, Glutamine (Q), (neutral, polar)	H (Histidine)	—	—	H	—	—
48	Glycine (G), (Nonpolar)	E	—	—	—	—	—
49	P, Proline	—	—	—	H	—	—
51	K, Lysine (K), (Basic, Polar)	E	—	—	—	N	—
52	Leucine (L), (Nonpolar)	M	M	M	M	M	M
57	Methionine (M). (Nonpolar)	I	I	I	I	I	I
65	I, Isoleucine	V	—	—	—	—	—
76	Glycine (G), (Nonpolar)	S	S	S	S	S	S
78	Leucine (L), (Nonpolar)	F	F	F	F	F	F
85	K, Lysine (K), (Basic, Polar)	—	—	—	—	—	R
93	E, Glutamic acid, (Acidic polar)	—	—	—	Q	—	—
96	Asparagine (N), (neutral, polar	S	S	S	S	S	S
99	Serine (S), (neutral, polar)	N	N	N	N	N	N
106	Arginine (R), (Basic, Polar)	—	—	—	—	T	T
108	V, Valine	—	—	—	E	—	—
156	I, Isoleucine	L	L	L	_	L	L
178	K, Lysine (K), (Basic, Polar)	E	E	E	_	E	E
179	A, Alanine	T	T	T	_	A	A
182	D, Aspartic acid. (Acidic polar)	E	E	E	_	E	E
184	V, Valine	I	I	I	_	I	I

^∗^Numbering is according to the reference sequence ACF49259.1.

### 3.4. Prediction of Secondary Structure

**FIGURE 6:**
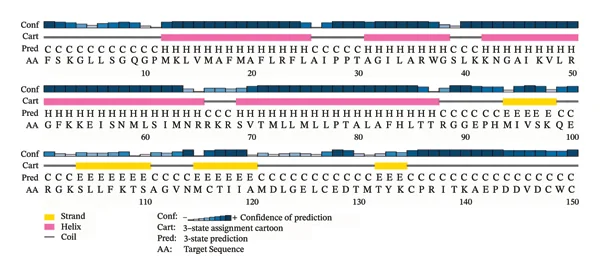
Predicted secondary structure of the reference DENV‐1 protein (accession: ACF49259). The secondary structure composition of the reference protein is illustrated using a symbolic representation indicating *α*‐helices, *β*‐sheets, and coils. The prediction was generated using a secondary structure prediction tool, where “H” denotes helices, “E” represents extended strands (beta‐sheets), and “C” refers to random coils. The legend also includes a confidence score for the prediction, highlighting regions with high reliability.

**FIGURE 7:**
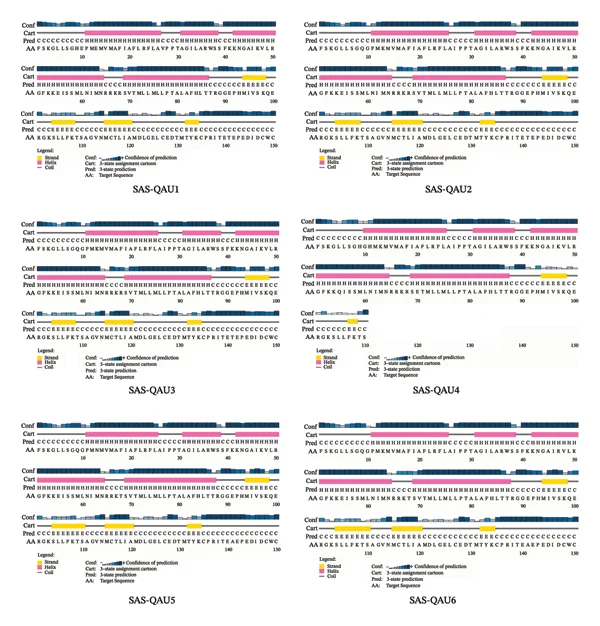
Predicted secondary structures of DENV‐1 isolates SAS‐QAU1–SAS‐QAU6. This figure displays the predicted secondary structural elements for each studied isolate, including *α*‐helices, *β*‐sheets, and coils. The visualization reveals structural conservation as well as regions of variation among the isolates. Color‐coded legends indicate the structural elements and prediction confidence scores, facilitating comparison with the reference protein. Variations in the secondary structure may correspond to amino acid substitutions observed in the primary sequences.

An online server SOPMA was employed for the prediction of the secondary structure of the reference protein CprM region of DENV1 under the accession number (AWV55256) and all the isolates (SAS‐QAU1, SAS‐QAU2, SAS‐QAU3, SAS‐QAU4, SAS‐QAU5, SAS‐QAU6) as shown in Figures 6 and 7. The server used bioinformatics approaches that attempt to determine the local secondary structures of proteins based only on their amino acid sequence. Reference structure protein has an alpha helix of 40.00%, beta sheets of 18.00%, and random coils of 42.00%. This shows that conserved regions are present, which maintain the secondary structure of the CprM protein. The secondary structure of DENV1 isolate QAU‐SAS 1 has an alpha helix of 39.33%, beta sheets of 18.00%, random coils of 42.67%, QAU‐SAS 2 has an alpha helix of 40.67%, beta sheets of 18.00%, and random coils of 41.33%, while the secondary structure of DENV1 isolates QAU‐SAS 3 has an alpha helix of 40.67%, beta sheets of 18.00%, random coils of 41.33%. The QAU‐SAS 4 has an alpha helix of 45.45%, beta sheets of 19.09%, and random coils of 35.45%. QAU‐SAS 5 has an alpha helix of 40.67%, beta sheets of 18.00%, and random coils of 41.33%. The secondary structure of isolate QAU‐SAS 6 has an alpha helix of 40.00%, beta sheets of 18.00%, and random coils of 42.00%, as shown in Table 3.

**TABLE 3:** Predicted secondary structure composition of DENV‐1 isolates and the reference sequence.

Isolate name	Accession no	Alpha helix	Beta sheets	Random coils
Reference	ACF49259	40.00%	18.00%	42.00%
SAS‐QAU1	PQ699316	39.33%	18.00%	42.67%
SAS‐QAU2	PQ699317	40.67%	18.00%	41.33%
SAS‐QAU3	PQ699318	40.67%	18.00%	41.33%
SAS‐QAU4	PQ699319	45.45%	19.09%	35.45%
SAS‐QAU5	PQ699320	40.67%	18.00%	41.33%
SAS‐QAU6	PQ699321	40.00%	18.00%	42.00%

*Note:* The table summarizes the percentage distribution of alpha helices, beta sheets, and random coils in the reference protein (ACF49259) and six DENV‐1 isolates (SAS‐QAU1–SAS‐QAU6). Secondary structure prediction was performed to evaluate structural conservation and variations among the sequences. Alpha helices, beta sheets, and coils are presented as a percentage of the total amino acid residues. Minor differences in the secondary structure content reflect sequence‐level substitutions that may influence protein folding and function.

### 3.5. Prediction of Tertiary Structure and Visualization

**FIGURE 8:**
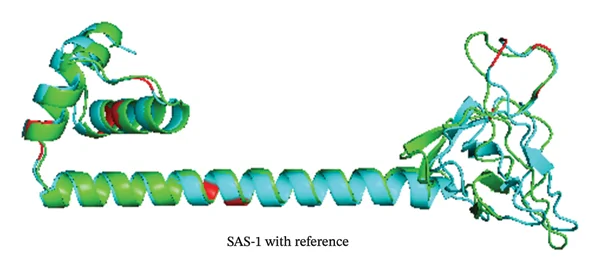
Structural alignment of isolate SAS‐QAU1 with the reference DENV‐1 protein. The superimposed 3D structures of SAS‐QAU1 and the reference DENV‐1 protein highlight their structural similarities and differences. The alignment was performed to assess structural conservation and deviations introduced by sequence mutations. Colored ribbons represent the overall protein fold, while substituted or mutated residues in SAS‐QAU1 are marked in red, indicating positions of potential functional or structural impact.

**FIGURE 9:**
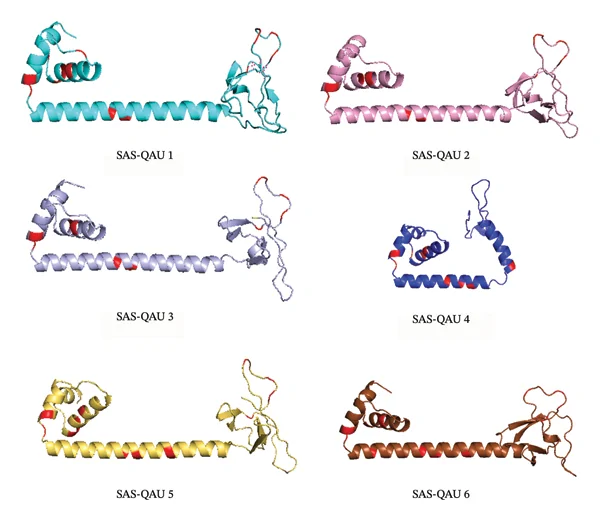
Predicted tertiary structures of DENV‐1 isolate SAS‐QAU1–SAS‐QAU6. The 3D models display the tertiary conformations of the DENV‐1 isolates generated through homology modeling. Each protein is shown using a distinct color scheme, with mutated or substituted amino acid residues highlighted in red. These structural visualizations provide insights into spatial rearrangements caused by sequence variability and their possible implications for antigenicity, receptor interaction, or protein stability.

The 3D structures of all six DENV‐1 isolates (SAS‐QAU1–SAS‐QAU6) were successfully modeled and compared to the reference DENV‐1 protein structure. The structure of SAS‐QAU1 closely aligned with the reference, indicating high structural similarity (Figure 8). In contrast, SAS‐QAU2–SAS‐QAU6 exhibited structural variations, with notable amino acid substitutions observed at specific positions. These substitutions, highlighted in red, reflect potential structural impacts and are shown in Figure 9. These differences may contribute to changes in antigenicity, stability, or protein function.

**FIGURE 10:**
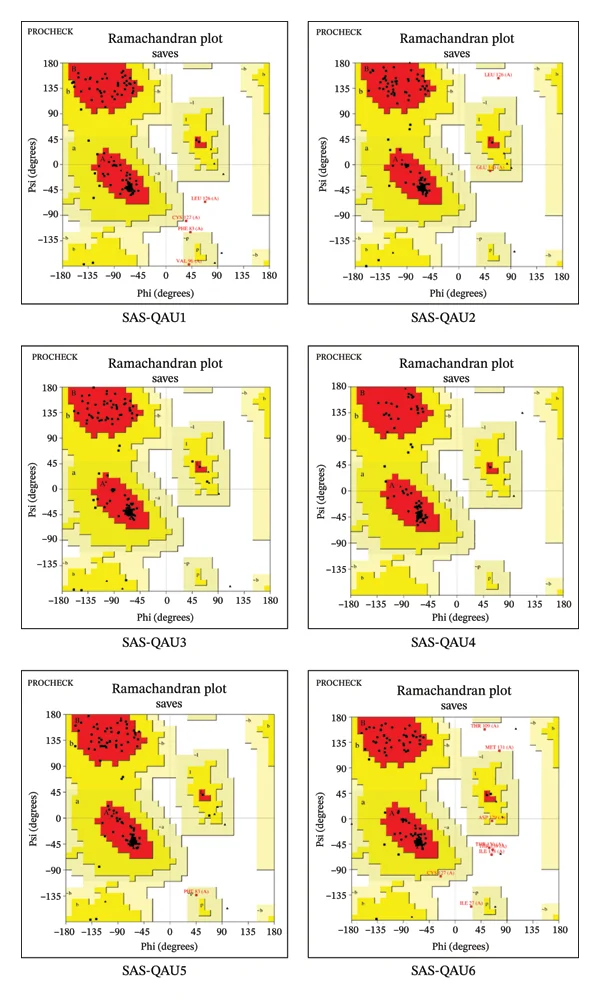
It shows the structural validation of the predicted protein models of dengue virus isolates (SAS‐QAU1–SAS‐QAU6) using Ramachandran plot and ERRAT analysis.

These tertiary structures were further evaluated using predicted protein models, which were validated using the Ramachandran plot analysis and ERRAT assessment, which is given in Figure 10. Most residues were located within favored regions of the Ramachandran plots, indicating acceptable stereochemical quality. The percentages of residues in favored regions were SAS‐QAU1: 90.1%, SAS‐QAU2:93.1%, SAS‐QAU3: 92.3%, SAS‐QAU4:94.7%, SAS‐QAU5: 92.3%, and SAS‐QAU6: 89.2%. Furthermore, ERRAT scores ranged from 91.11 to 100, supporting the reliability of the generated models of our studied isolates.

### 3.6. Evaluation of the Antigenicity and Immunogenicity of Isolated Proteins

Among the six isolates studied, SAS‐QAU4 and SAS‐QAU6 exhibited a slightly reduced antigenicity compared to the reference DENV‐1 protein. In contrast, the remaining isolates (SAS‐QAU1, SAS‐QAU2, SAS‐QAU3, and SAS‐QAU5) showed a minor increase in antigenic potential. Regarding immunogenicity, isolates SAS‐QAU1, SAS‐QAU2, SAS‐QAU3, and SAS‐QAU5 demonstrated a reduced probability of immune recognition (66%) relative to the reference (100%). SAS‐QAU4 and SAS‐QAU6 were predicted to be nonimmunogenic as shown in Table 4.

**TABLE 4:** Antigenicity and immunogenicity profiles of DENV‐1 isolates (SAS‐QAU1–SAS‐QAU6) compared with the reference protein (ACF49259.1).

S No	Isolates	Protein sequences	Antigenicity score	Immunogenicity
1	Reference Protein (ACF49259.1)	FSKGLLSGQGPMKLVMAFMAFLRFLAIPPTAGILARWGSLKKNGAIKVLRGFKKEISNMLSIMNRRKRSVTMLLMLLPTALAFHLTTRGGEPHMIVSKQERGKSLLFKTSAGVNMCTIIAMDLGELCEDTMTYKCPRITKAEPDDVDCWC	0.4988 (Probable antigen).	Predicted immunogen (probability 100%)
2	SAS‐QAU1	FSKGLLSGHEPMEMVMAFIAFLRFLAVPPTAGILARWSSFKKNGAIKVLRGFKKEISSMLNIMNRRKRSVTMLLMLLPTALAFHLTTRGGEPHMIVSKQERGKSLLFKTSAGVNMCTLIAMDLGELCEDTMTYKCPRITETEPEDIDCWC	0.5016 (Probable antigen).	Predicted immunogen (probability 66%)
3	SAS‐QAU2	FSKGLLSGQGPMKMVMAFIAFLRFLAIPPTAGILARWSSFKKNGAIKVLRGFKKEISSMLNIMNRRKRSVTMLLMLLPTALAFHLTTRGGEPHMIVSKQERGKSLLFKTSAGVNMCTLIAMDLGELCEDTMTYKCPRITETEPEDIDCWC	0.5100 (Probable antigen).	Predicted immunogen (probability 66%)
4	SAS‐QAU3	FSKGLLSGQGPMKMVMAFIAFLRFLAIPPTAGILARWSSFKKNGAIKVLRGFKKEISSMLNIMNRRKRSVTMLLMLLPTALAFHLTTRGGEPHMIVSKQERGKSLLFKTSAGVNMCTLIAMDLGELCEDTMTYKCPRITETEPEDIDCWC	0.5100 (Probable antigen).	Predicted immunogen (probability 66%)
5	SAS‐QAU4	FSKGLLSGHGHMKMVMAFIAFLRFLAIPPTAGILARWSSFKKNGAIKVLRGFKKQISSMLNIMNRRKRSETMLLMLLPTALAFHLTTRGGEPHMIVSKQERGKSLLFKTS	0.4252 (Probable antigen).	Predicted nonimmunogen (probability 100%)
6	SAS‐QAU5	FSKGLLSGQGPMNMVMAFIAFLRFLAIPPTAGILARWSSFKKNGAIKVLRGFKKEISSMLNIMNRRKTSVTMLLMLLPTALAFHLTTRGGEPHMIVSKQERGKSLLFKTSAGVNMCTLIAMDLGELCEDTMTYKCPRITEAEPEDIDCWC	0.5087 (Probable antigen).	Predicted immunogen (probability 66%)
7	SAS‐QAU6	FSKGLLSGQGPMKMVMAFIAFLRFLAIPPTAGILARWSSFKKNGAIRVLRGFKKEISSMLNIMNRRKTSVTMLLMLLPTALAFHLTTRGGEPHMIVSKQERGKSLLFKTSAGVNMCTLIAMDLGELCEDTMTYKCPRITEAEPEDIDCWC	0.4896 (Probable antigen).	Predicted nonimmunogen (probability 66%)

*Note:* This table presents the predicted antigenic and immunogenic characteristics of the DENV‐1 isolates studied. Each entry includes the protein sequence, antigenicity score, and immunogenicity probability. Antigenicity was assessed using the VaxiJen v2.0 server, with scores above 0.4 considered as probable antigens. Immunogenicity was predicted based on the ability of each sequence to stimulate an immune response, presented as a binary prediction (probable immunogen or nonimmunogen) along with the corresponding probability value. The reference protein serves as a benchmark for comparative analysis. Minor amino acid variations in isolates may account for observed differences in immunogenic potential.

### 3.7. ADMET Results

From the initial pool of phytochemicals, 15 compounds were identified as having favorable drug‐like properties based on ADMET evaluation. These compounds demonstrated high GI absorption, no BBB permeability, and zero Lipinski’s rule violations and were classified as nontoxic and noncarcinogenic. The top hit 9 out of 15 phytochemicals were further analyzed based on the above characteristics, which include Limonin, Mundulinol, Isopomiferin, Silydianin, Derrisin, Andrographolide, Apigenin, Chrysoeriol, and Moringin, extracted from various medicinal plants such as *Azadirachta indica*, *Mundulea sericea*, *Maclura pomifera*, *Silybum marianum*, *Derris elliptica*, *Andrographis paniculata*, and *Moringa oleifera*. Their molecular formulas, source plants, and chemical structures are summarized in Table 5, while their ADMET profiles are detailed in Table 6.

**TABLE 5:** List of selected phytochemicals with their molecular formula, plant sources, and 2D chemical structures.

Compound	Formula	Plant	Structure
Limonin	C_26_H_30_O_8_	*Azadirachta indica*	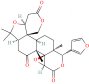
Mundulinol	C_25_H_26_O_5_	*Mundulea sericea*	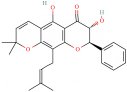
Isopomiferin	C_25_H_24_O_6_	*Maclura pomifera*	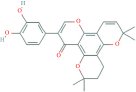
Silydianin	C_25_H_22_O_10_	*Silybum marianum*	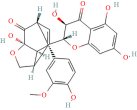
Derrisin	C_23_H_24_O_8_	*Derris elliptica*	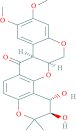
Andrographolide	C_20_H_30_O_5_	*Andrographis paniculate*	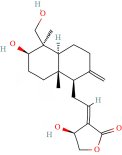
Apigenin	C_15_H_10_O_5_	*Andrographis paniculata*	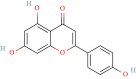
Chrysoeriol	C_16_H_12_O_6_	*Andrographis paniculata*	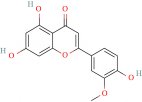
Moringin	C_14_H_17_NO_5_S	*Moringa oleifera*	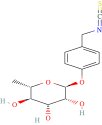

*Note:* The table presents nine phytochemicals evaluated for their potential interaction with the SAS‐QAU1 protein through molecular docking studies. Each entry includes the compound name, molecular formula, and the plant species from which the compound is derived. The rightmost column shows the 2D structural representation of each phytochemical as obtained from chemical databases or molecular drawing tools. These compounds were selected based on their reported pharmacological activities and relevance to antiviral or immunomodulatory research.

**TABLE 6:** Predicted absorption, distribution, metabolism, excretion, and toxicity (ADMET) profiling of selected phytochemicals.

Compound	Plant	Estimated solubility log score	Estimated solubility class	Gastrointestinal absorption	Blood–brain barrier penetration	Lipinski violation	Toxicity	Carcinogenicity
Limonin	Azadirachta indica	−3.92	Soluble	High	No	Yes; 0 violation	Non‐toxic	Noncarcinogenic
Mundulinol	Mundulea sericea	−5.86	Moderate soluble	High	No	Yes; 0 violation	Non‐toxic	Noncarcinogenic
Isopomiferin	Maclura pomifera	−5.52	Moderate soluble	High	No	Yes; 0 violation	Non‐toxic	Noncarcinogenic
Silydianin	Silybum marianum	−3.39	Soluble	High	No	Yes; 0 violation	Non‐toxic	Noncarcinogenic
Derrisin	Derris elliptica	−3.72	Soluble	High	No	Yes; 0 violation	Non‐toxic	Noncarcinogenic
Andrographolide	Andrographis paniculata	−3.18	Soluble	high	No	Yes; 0 violation	Non‐toxic	Noncarcinogenic
Apigenin	Andrographis paniculata	−3.94	Soluble	high	No	Yes; 0 violation	Non‐toxic	Noncarcinogenic
Chrysoeriol	Andrographis paniculata	−4.06	Moderate soluble	high	No	Yes; 0 violation	Non‐toxic	Noncarcinogenic
Moringin	Moringa oleifera	−2.54	Soluble	high	No	Yes; 0 violation	Non‐toxic	Noncarcinogenic

*Note:* This table summarizes the pharmacokinetic and toxicity‐related properties of nine phytochemicals assessed in the study. Parameters include estimated solubility (log S) and solubility classification, predicted gastrointestinal (GI) absorption, blood–brain barrier (BBB) penetration potential, compliance with Lipinski’s rule of five, toxicity, and carcinogenicity. All compounds demonstrated high GI absorption and no BBB penetration. None violated Lipinski’s rules, indicating good drug‐likeness. Additionally, all compounds were predicted to be nontoxic and noncarcinogenic, supporting their potential for further drug development studies.

### 3.8. Molecular Docking

**FIGURE 11:**
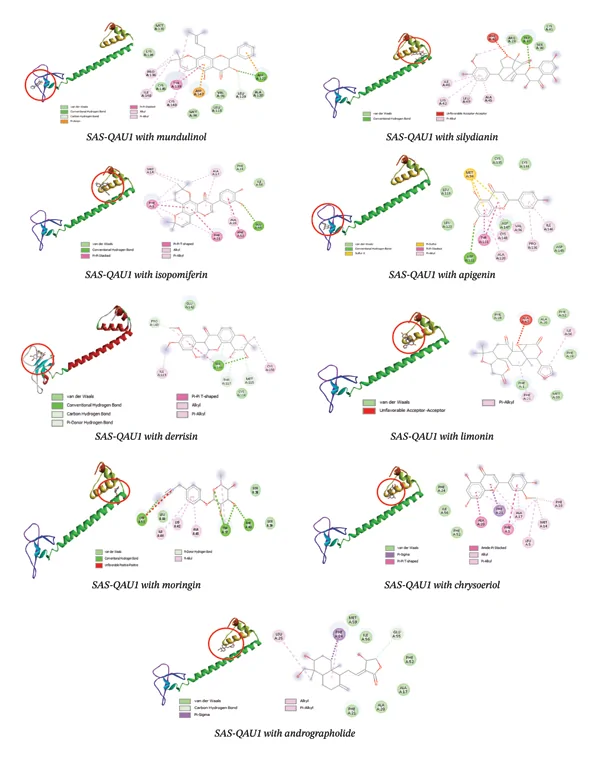
Molecular docking interactions of SAS‐QAU1 with selected phytochemicals. The image illustrates the binding interactions between the modeled SAS‐QAU1 protein and nine phytochemicals: Mundulinol, Silydianin, Isopomiferin, Apigenin, Derrisin, Limonin, Moringin, Chrysoeriol, and Andrographolide. Each panel includes a 3D representation of the ligand–protein complex and a 2D interaction diagram detailing specific bonding interactions. Interaction types are denoted by colored lines and symbols: green for van der Waals forces, pink for Pi–Pi stacking and Pi–alkyl interactions, yellow for conventional hydrogen bonds, and red for unfavorable interactions. Amino acid residues involved in binding are labeled and color‐coded by interaction type. These visualizations help elucidate the binding modes and key residues contributing to the stabilization of each ligand within the SAS‐QAU1 active site.

Due to the high sequence identity among all isolates, SAS‐QAU1 was selected as the representative sequence for downstream structural modeling and docking studies because of the high sequence similarity among our isolates. Docking simulations revealed that the selected phytochemicals displayed varying interaction profiles with the CprM junction region of isolate SAS‐QAU1. Among the 15 compounds evaluated, 9 phytochemicals exhibited docking scores greater than −100, indicating strong binding affinity and potential inhibitory activity against DENV‐1. These include Limonin, Mundulinol, Isopomiferin, Silydianin, Derrisin, Andrographolide, Apigenin, Chrysoeriol, and Moringin. The docking score, confidence score, and ligand RMSD (Å) values for each ligand–protein complex are summarized in Table 7. Notably, Silydianin exhibited the strongest interaction, with a docking score of −159.81, followed by Isosilybin and Isopomiferin. Molecular docking of SAS‐QAU1 with nine phytochemicals revealed diverse noncovalent interactions, including hydrogen bonds, van der Waals forces, and Pi‐type contacts. These interactions stabilize ligand binding within the active site and suggest therapeutic relevance through structural compatibility, as shown in Figure 11.

**TABLE 7:** Molecular docking scores, confidence scores, and ligand RMSD values for selected phytochemical–SAS‐1 protein complexes.

Docking complex	Docking score	Confidence score	Ligand rmsd (Å)
SAS‐1 with Derrisin	−132.85	0.4151	48.71
SAS‐1 with Isosilybin	−148.70	0.4935	51.73
SAS‐1 with Silydianin	−159.81	0.5489	24.35
SAS‐1 with Isopomiferin	−137.86	0.4396	11.43
SAS‐1 with Mundulinol	−127.69	0.3903	62.27
SAS‐1 with Andrographolide	−113.60	0.3256	16.66
SAS‐1 with apigenin	−109.44	0.3076	60.43
SAS‐1 with Chrysoeriol	−112.81	0.3222	12.94
SAS‐1 with Moringin	−112.45	0.3206	25.18
SAS‐1 with Limonin	−134.93	0.4252	13.22

*Note:* A total of nine phytochemical ligands were assessed for bounding to the SAS‐1 protein. The docking score (in kcal/mol) reflects the binding affinity, with more negative values indicating stronger predicted interactions. The confidence score represents the reliability of the predicted binding pose. Ligand RMSD (root‐mean‐square deviation, in Ångströms) indicates the structural deviation between the docked and reference conformations of the ligand, with lower values signifying higher pose accuracy. These values collectively help assess the binding strength and stability of each phytochemical–protein complex.

## 4. Discussion

DENV is a globally significant arboviral pathogen, responsible for an estimated 390 million infections annually, with considerable public health impact in tropical and subtropical regions [32]. In Pakistan, dengue has become endemic, with increasingly frequent outbreaks reported across all provinces since the first major epidemic in 1994 [33]. The current study was conducted to investigate the molecular characteristics of DENV‐1 strains circulating during an outbreak in Peshawar, Pakistan. Specifically, we aimed to determine the prevailing serotype, analyze genetic variability in the CprM junction region, assess antigenicity and immunogenicity, and evaluate phytochemicals as potential antiviral candidates. To our knowledge, this is one of the first studies to evaluate the CprM junction region of DENV‐1 isolates from Pakistan in conjunction with a docking‐based phytochemical screening pipeline.

The sequenced PCR‐positive isolates in our study were confirmed to belong to serotype DENV‐1 in the sampled outbreak cohort from Peshawar. Phylogenetic analysis based on the CprM junction region revealed that the studied isolates (SAS‐QAU1–SAS‐QAU6) clustered closely with reference strains previously reported from China, India, Bangladesh, and Pakistan. This clustering suggests close genetic relatedness within the established DENV‐1 lineage and may reflect regional circulation or transboundary movement of related strains. Such phylogenetic relationships highlight the importance of continued molecular surveillance for understanding regional dengue dynamics [34]. Although DENV‐2 has frequently been reported as a predominant serotype in several dengue outbreaks in Pakistan and may show greater epidemic potential in some settings [11, 12], the detection of DENV‐1 in the present outbreak cohort remains epidemiologically relevant, particularly in the context of serotype cocirculation and limited molecular surveillance in Pakistan [13–15]. Moreover, amino acid substitutions within the CprM structural region may contribute to the predicted antigenicity and immunogenicity variations observed among the DENV‐1 isolates in this study.

Multiple amino acid substitutions were identified in the CprM region of the DENV‐1 isolates when aligned with the reference polyprotein sequence ACF49259.1. Key substitutions included Q47H, G48E, K51 E/N, L52 M, M57I, and other changes distributed across two functionally relevant regions. Substitutions located between positions 47 and 108 fall mainly within the capsid/C‐prM junction region [35, 36]. The DENV capsid protein plays a crucial role in viral RNA packaging, genome compaction, lipid‐droplet association, and formation of infectious virions [35]. Therefore, substitutions such as Q47H, G48E, K51 E/N, L52 M, M57I, G76 S, L78F, K85R, N96 S, S99N, R106 T, and V108E may potentially influence local charge distribution, hydrophobic interactions, capsid stability, or virion assembly–related functions [32, 37]. In contrast, substitutions observed at positions 156–184 fall within the pr peptide region of prM [36, 38]. The pr peptide acts as a protective cap over the envelope protein fusion loop during virion maturation, thereby preventing premature fusion before viral release [38]. Therefore, substitutions such as I156 L, K178E, A179 T, D182E, and V184I may have possible implications for prM‐E interaction, maturation efficiency, and antigenic recognition. Since prM/pr‐containing immature or partially mature dengue virions can be recognized by cross‐reactive and sometimes subneutralizing antibodies, mutations in this region may also influence antibody‐mediated immune responses, including antibody‐dependent enhancement‐related effects [39–43]. However, these interpretations remain predictive, and further infectivity assays, neutralization assays, and epitope‐mapping studies are required to confirm the biological consequences of these substitutions.

Assessment of antigenicity and immunogenicity revealed notable variability among the DENV‐1 isolates. Compared to the reference sequence ACF49259.1, four isolates, SAS‐QAU1, SAS‐QAU2, SAS‐QAU3, and SAS‐QAU5, showed a marginal increase in antigenicity scores, while SAS‐QAU4 and SAS‐QAU6 exhibited slightly reduced antigenic potential. Immunogenicity predictions showed that SAS‐QAU1, SAS‐QAU2, SAS‐QAU3, and SAS‐QAU5 had reduced immune recognition probability, whereas SAS‐QAU4 and SAS‐QAU6 were predicted to be nonimmunogenic. These variations may be associated with amino acid substitutions occurring in functionally or antigenically relevant regions of the CprM segment, potentially altering epitope presentation or immune recognition. However, these computational predictions require further validation through in vitro neutralization assays and T‐cell epitope mapping [44].

In the absence of specific antiviral therapies for dengue, the study evaluated the therapeutic potential of plant‐derived phytochemicals targeting the CprM region of DENV‐1. From an initial pool of 60 candidates, 15 compounds met ADMET and drug‐likeness criteria, including high GI absorption, no BBB permeability, noncarcinogenicity, and zero Lipinski’s rule violations [45–47]. The CprM region has been explored as a potential antiviral target because of its sequence conservation, essential role in virion assembly and maturation, and suitability for broad‐spectrum antiviral intervention [48]. Molecular docking revealed that nine phytochemicals, Limonin, Mundulinol, Isopomiferin, Silydianin, Derrisin, Andrographolide, Apigenin, Chrysoeriol, and Moringin, exhibited strong binding affinity (docking scores < −100) with SAS‐QAU1. Silydianin demonstrated the highest binding strength (−159.81), suggesting a stable and potentially inhibitory interaction. These findings align with previous studies that identified several of these compounds as inhibitors of viral proteins [49] and extend their relevance to the structurally conserved CprM region, supporting their potential utility as dengue antivirals. These compounds may interfere with viral replication or capsid stability by targeting structurally conserved residues in the CprM junction region, though further validation is needed. Detailed docking interactions and ligand–protein complex visualizations are presented in Table 7 and Figure 10.

A major strength of this study is the integrated molecular and computational assessment of DENV‐1 isolates circulating in Peshawar, Pakistan, including the identification of amino acid substitutions in the CprM region, evaluation of their possible antigenic and immunogenic relevance, and prioritization of potential phytochemical inhibitors through docking‐based screening. However, certain limitations must be acknowledged. The analysis was limited to a small number of sequenced isolates, and only the CprM junction region was examined rather than the complete viral genome. In addition, antigenicity, immunogenicity, structural modeling, and docking analyses were based on computational predictions. Although these approaches provide useful preliminary insights, they cannot fully represent protein flexibility, biological complexity, true binding affinity, or antiviral activity under physiological conditions. Future studies should include larger sample sizes, whole‐genome sequencing, expanded surveillance across multiple regions and serotypes, cell‐based antiviral assays, infectivity assays, neutralization assays, and epitope‐mapping experiments to validate the biological relevance of the observed mutations and the antiviral potential of the shortlisted phytochemicals.

## 5. Conclusion

This study provides a comprehensive molecular characterization of DENV‐1 isolates from the dengue outbreak in Peshawar, Pakistan. Phylogenetic analysis revealed that the isolates form a distinct subclade within the DENV‐1 lineage, indicating regional viral evolution and potential transboundary transmission. Several amino acid substitutions were identified in the CprM junction region, some of which may affect viral structure, antigenicity, and immune recognition. In silico predictions demonstrated considerable antigenic and immunogenic variability among the isolates, with two strains predicted to be nonimmunogenic, potentially impacting diagnostic accuracy and vaccine responsiveness. Importantly, virtual screening of phytochemicals identified nine plant‐derived compounds with favorable ADMET properties and strong binding affinity to the CprM region, suggesting their promise as antiviral candidates. These findings not only enhance our understanding of the molecular evolution and immunological landscape of DENV‐1 but also propose a novel integrative molecular computational framework for identifying therapeutic agents. To further substantiate these findings, a comprehensive functional study, including cell‐based assays and molecular dynamics simulations, are recommended to validate the efficacy and mechanism of action of these phytochemicals.

## Author Contributions

Sajid Ali Shah and Muhammad Ali: conceptualized the study; Sajid Ali Shah: sample collection and data collection; Sajid Ali Shah and Syed Ahsan Shahid: methodology, laboratory analysis, software analysis, and data curation; Sajid Ali Shah, Syed Ahsan Shahid, and Sidra Rahman: writing–original draft preparation; Sidra Rahman and Muhammad Ali: writing–reviewing and editing; Muhammad Ali: supervision.

## Funding

We are grateful to Professor Zengli Shi, Wuhan Institute of Virology, for providing technical support. This work was financially supported by the Alliance of International Science Organization (Grant ID: ANSO‐CR‐PP‐2021‐05).

## Ethics Statement

The Bio‐Ethical Committee (BEC‐FBS‐QAU2023‐520) at Quaid‐i‐Azam University, Islamabad, approved this study. Participants provided informed consent to participate in the study and no personal or identifiable data were collected or shared.

## Conflicts of Interest

The authors declare no conflicts of interest.

## Data Availability

The current study sequences are deposited in GenBank database under the accession numbers PQ699316, PQ699317, PQ699318, PQ699319, PQ699320, and PQ699321, which is represented in the study as SAS‐QAU1, SAS‐QAU2, SAS‐QAU3, SAS‐QAU4, SAS‐QAU5, and SAS‐QAU6, respectively. Further, the data will be available from the corresponding author upon reasonable request.
